# Structural and Pharmacological Insights into Propranolol: An Integrated Crystallographic Perspective

**DOI:** 10.3390/ijms262010080

**Published:** 2025-10-16

**Authors:** Adrianna Witczyńska, Łukasz Fijałkowski, Dagmara Mirowska-Guzel, Kamila Blecharz-Klin, Alicja Nowaczyk

**Affiliations:** 1 Department of Organic Chemistry, Faculty of Pharmacy, Collegium Medicum in Bydgoszcz, Nicolaus Copernicus University, 87-100 Toruń, Poland; adrianna.witczynska@doktorant.umk.pl (A.W.); l.fijalkowski@cm.umk.pl (Ł.F.); 2 Department of Experimental and Clinical Pharmacology, Centre for Preclinical Research and Technology CePT, Medical University of Warsaw, Centre for Preclinical Research and Technology, Banacha 1b, 02-097 Warsaw, Poland; dagmara.mirowska-guzel@wum.edu.pl (D.M.-G.); kamilla.blecharz-klin@wum.edu.p (K.B.-K.)

**Keywords:** propranolol, human β_2_-adrenergic receptor, Cel7A enzyme, lactoferrin

## Abstract

Propranolol is a non-selective β-adrenergic receptor antagonist widely used in cardiovascular and neurological therapy. Its naphthalene-based structure contributes to its high lipophilicityand central nervous system penetration. Clinically, propranolol is indicated for hypertension, arrhythmias, anxiety, migraine, and other conditions. It undergoes extensive hepatic metabolism via cytochrome P450 enzymes, notably CYP2D6, with a significant first-pass effect limiting oral bioavailability. This review integrates pharmacological profiling with crystallographic analysis to explore propranolol’s molecular interactions and therapeutic versatility. High-resolution crystal structures of the human β_2_-adrenergic receptor (hβ_2_-AR), particularly PDB ID: 6PS5 obtained via serial femtosecond crystallography (SFX), reveal key binding determinants responsible for receptor affinity and antagonism. Comparative structural analysis with other β-blockers—alprenolol, timolol, and carvedilol—highlights how variations in aromatic and heterocyclic frameworks influence pharmacokinetics and receptor selectivity. Superimposition results (RMSD: 0.032 for propranolol–alprenolol, 0.078 for propranolol–carvedilol, and 1.078 for propranolol–timolol) quantitatively illustrate molecular similarity and divergence. The enantioselective behavior of propranolol is also discussed, with the S-enantiomer showing greater receptor affinity and pharmacological potency than the R-form. Beyond canonical β-adrenergic targets, propranolol interacts with non-canonical proteins such as the cellulase enzyme Cel7A and lactoferrin, suggesting off-target effects and novel therapeutic potential. These findings underscore the importance of propranolol’s amphiphilic character, stereochemistry, and electrostatic properties in shaping its pharmacological profile. Overall, the integration of crystallographic data with pharmacological insights supports the rational design of next-generation β-adrenergic ligands with enhanced selectivity, bioavailability, and clinical efficacy.

## 1. Introduction

Propranolol, a pioneering β-adrenergic receptor (β-AR) antagonist, was developed by Sir James Black in the 1960s [1,2], revolutionizing the treatment landscape for cardiovascular diseases such as angina pectoris and hypertension. Over the decades, its clinical applications have expanded significantly, including its use in treating neurological and psychiatric disorders. The present study aims to provide a comprehensive review of propranolol’s chemical, pharmacological, and therapeutic properties, emphasizing its broad clinical relevance in modern medicine. Initially introduced for angina and hypertension, propranolol’s therapeutic indications have since expanded to include neurological and off-label applications. It is widely used in the management of hypertension, supraventricular and ventricular arrhythmias, and secondary prevention following myocardial infarction [3]. Propranolol has also gained approval for the prophylaxis of migraine and essential tremor [4], as well as off-label uses such as managing performance anxiety and stage fright, where its capacity to attenuate the sympathetic nervous system’s response is particularly beneficial [5]. In the treatment of hyperthyroidism, propranolol is frequently used to manage thyrotoxicosis symptoms such as palpitations and tremors, due to its beta-blocking and anti-sympathetic properties [6]. The use of propranolol in infantile hemangiomas has also emerged as a successful treatment, though the exact mechanism of action remains under investigation [7].

This review introduces an innovative interdisciplinary perspective by integrating pharmacological profiling with crystallographic characterization of propranolol. In contrast to previous studies, it examines enantioselective interactions and off-target binding to non-canonical proteins such as Cel7A and lactoferrin, thereby offering a multidimensional framework that may inform the future design of β-blockers.

## 2. Historical Development and Pharmacological Profile of Propranolol

### 2.1. Mechanism of Action

Propranolol is a non-selective β-AR receptor antagonist that blocks both β_1_ and β_2_ receptors, thereby inhibiting the physiological effects of catecholamines such as adrenaline and noradrenaline [8]. These catecholamines normally stimulate the sympathetic nervous system, leading to increased heart rate, contractility, and bronchodilation. By competitively binding to β-receptors, propranolol reduces cardiac output and suppresses sympathetic nervous system activity [3]. At higher concentrations, propranolol exhibits membrane-stabilizing activity, which may contribute to its antiarrhythmic effects [9]. Its lack of intrinsic sympathomimetic activity renders it contraindicated in patients with asthma or severe bradycardia due to the risk of bronchospasm. Additionally, propranolol’s lipophilicity allows it to cross the blood–brain barrier, enabling central nervous system (CNS) effects such as anxiolysis and migraine prophylaxis [10].

### 2.2. Clinical Application

Propranolol is indicated for a broad spectrum of cardiovascular and neurological conditions. It is commonly prescribed for hypertension, angina pectoris (excluding Prinzmetal’s variant), supraventricular and ventricular arrhythmias, and for the secondary prevention of myocardial infarction [6]. In cardiomyopathies, propranolol contributes to reducing left ventricular outflow tract obstruction and improve diastolic filling. It is also used in the perioperative management of pheochromocytoma, in combination with α-adrenergic blockers to prevent hypertensive crises [11]. From a neurological perspective, propranolol is effective in the management of essential tremor, migraine prophylaxis, and anxiety disorders, particularly those accompanied by pronounced somatic symptoms. It has shown efficacy in reducing performance anxiety and situational stress responses [12]. In gastroenterology, propranolol is used to prevent upper gastrointestinal bleeding in patients with portal hypertension and esophageal varices by reducing portal venous pressure [13]. Its versatility in clinical applications is supported by its pharmacokinetic properties, including rapid absorption, hepatic metabolism, and variable bioavailability depending on formulation and route of administration.

### 2.3. Regulatory Milestones

Propranolol was first synthesized by Sir James Black in the early 1960s, marking a pivotal advancement in cardiovascular pharmacotherapy. It was approved for the treatment of angina pectoris in 1965, with its initial European registration occurring on 23 April 1964. The European Medicines Agency (EMA) subsequently approved propranolol for hypertension, angina pectoris, and cardiac arrhythmias [14]. In 1973, it received approval for essential tremor, and in 1976, its indications were expanded to include migraine prophylaxis. In 1987, propranolol was sanctioned for the treatment of anxiety disorders, including generalized anxiety disorder and situational anxiety [15]. Recent regulatory reviews have acknowledged propranolol’s extended therapeutic potential, including its off-label use in conditions such as infantile hemangiomas and post-traumatic stress disorder (PTSD), reflecting its evolving role in modern medicine [16].

## 3. Chemical Aspects of Propranolol

### 3.1. Propranolol’s Molecular Structure

**Figure 1:**
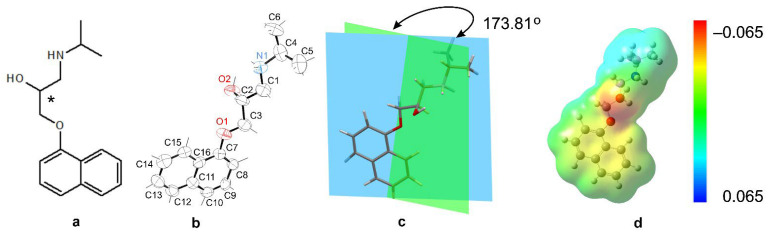
Structural and electrostatic characterization of propranolol. (**a**) Two-dimensional molecular diagram showing atomic connectivity and functional groups, including the secondary amine (–NH–), hydroxyl (–OH), and naphthalene ring system. (**b**) Three-dimensional ORTEP representation (CCDC code: 2262691) illustrating the spatial conformation and highlighting the chiral center at C2, which differentiates the (R)- and (S)-enantiomers. (**c**) Dihedral angle measurement (173.81°) between molecular planes, indicating near-planarity and potential for π-conjugation. (**d**) Electrostatic potential surface map ranging from −0.065 (red, electron-rich regions) to +0.065 (blue, electron-deficient regions), visualizing charge distribution relevant to hydrogen bonding, receptor binding, and amphiphilic behavior.

Propranolol’s (C_16_H_21_NO_2_) molecular structure is composed of a naphthalene ring system connected to a propanolamine side chain, which plays a critical role in the compound pharmacological activity. Figure 1 presents a comprehensive analysis of the propranolol molecule from its 2D structure, through to its 3D conformation and geometric analysis, to its electrostatic properties.

The core structure features two key functional groups, a secondary alcohol (–OH) and a secondary amine (–NH–), both attached to a hydrophobic naphthalene ring via an ester (–COOR) group (Figure 1). A nearly planar structure, which enhances π-conjugation and electronic delocalisation, is common in bioactive compounds, including both pharmaceuticals and natural products. This geometry may reduce conformational entropy by favoring stable binding conformations in biological environments. The –NH group serves as both a hydrogen bond donor and a nucleophilic site. –OH is capable of hydrogen bonding and increasing hydrophilicity; –COOR is a polar group that may undergo hydrolysis or participate in dipole interaction. These features suggest the molecule is amphiphilic with both hydrophilic and hydrophobic regions, which is relevant for solubility and membrane permeability. The amphiphilic character allows propranolol to interact with both hydrophobic and hydrophilic environments in the body, facilitating receptor binding and membrane penetration [2,9]. The asterisk-marked carbon atom in propranolol’s molecular structure is found at the C2 position, bonding to the hydroxyl (–OH) functional group. This location indicates the existence of a chiral center, which produces two enantiomers: (R)-propranolol and (S)-propranolol [17]. The existence of chirality has a substantial impact on the molecule’s physicochemical properties and pharmacological profile. Propranolol is synthesized as a racemic mixture, meaning it contains both the (S)- and (R)-enantiomers. In the context of electrostatic analysis (Figure 1d), the asterisk-marked carbon atom is crucial in shaping the molecule’s electrostatic potential distribution. Its close proximity to polar functional groups promotes localized electron density, which is required for intermolecular interactions, including hydrogen bonding and electrostatic interactions at receptor binding sites. This emphasizes the atom’s role not just in stereochemical definitions but also in altering the molecule’s interaction environment. Enantiomeric separation and the potential for enantiomer-specific effects are key considerations in drug metabolism and therapeutic efficacy, although clinical preparations of propranolol typically contain both enantiomers. Moreover, differences in enantiomeric configuration may lead to distinct side effect profiles [18].

### 3.2. Pharmacokinetic Properties of Propranolol

Table 1 summaries the key pharmacokinetic properties of propranolol, emphasizing its rapid metabolism and relatively short half-life.

The (S)-enantiomer primarily mediates β-blocking function, whereas the (R)-enantiomer has greatly reduced activity at adrenergic receptors (ARs). The stereoselectivity of propranolol is significant in its pharmacodynamics, as the (S)-enantiomer has a greater affinity for β-ARs [18]. The R-enantiomer has minimal β-blocking effects, but may still influence CNS activity. Propranolol is rapidly absorbed, but undergoes extensive enzymatic processing in the liver, resulting in low systemic availability (~26%). Metabolic transformation via cytochrome P450 enzymes (notably CYP2D6) produces active metabolites such as 4-hydroxypropranolol. It binds strongly to plasma proteins and has a short half-life (3–6 h), necessitating multiple daily doses. Its large volume of distribution (~3–5 L/kg) reflects extensive tissue penetration, especially in lipid-rich organs such as the brain and liver.

### 3.3. Propranolol’s Physicochemical Properties

A notable chemical property of propranolol is its lipophilicity, attributed to a lipophilic aromatic ring and an aliphatic chain, enabling its solubility in fatty tissues. The lipophilicity of a molecule is determined by the balance between hydrophobic and polar or ionizable groups. This property is essential since the gastrointestinal tract and various body fluids are aqueous, but many active pharmaceutical targets, such as transporters and receptors, rely on hydrophobic interactions for their effectiveness. The LogP of propranolol is 3.48 [20,21]. This suggests that propranolol exhibits a considerable propensity to partition into lipid environments rather than water, indicating substantial lipophilicity. Due to its lipophilicity, propranolol readily crosses the blood–brain barrier (BBB), which helps explain why some patients experience symptoms associated with the CNS, like drowsiness, vivid nightmares, or exhaustion [20]. The lipophilic characteristics of propranolol influence its tissue distribution, leading to a substantial volume of distribution (about 4 L/kg) [19].

Propranolol functions as a weak base, reflecting its ability to accept protons (H^+^) in aqueous solution. The pKa of propranolol is approximately 9.53, suggesting that in solutions with a pH lower than this value, propranolol predominantly exists in ionized form, whereas at higher pH values it remains largely non-ionized [22]. Propranolol’s weakly basic amine group means that it is primarily ionized at physiological pH. The charged and uncharged functionalities of propranolol facilitate specific interactions with diverse biological components, such as the charged surface and hydrophobic interior of bilayers. The unprotonated form exhibits high permeability, allowing for rapid diffusion across biological membranes, whereas the protonated form demonstrates at least an order of magnitude lower permeability, resulting in significantly slower membrane diffusion rates. In acidic environments, such as the stomach, propranolol predominantly exists in its protonated form, which exhibits reduced permeability across lipid membranes. In neutral or basic environments, such as the small intestine or bloodstream, a greater proportion remains unprotonated, facilitating more rapid and efficient membrane diffusion. The sensitivity to pH influences oral bioavailability, tissue penetration, and the onset of action. A 2024 study investigated the interaction of propranolol with biomimetic micellar systems, demonstrating that partitioning behavior is dependent on pH and influenced by the drug’s charge and lipophilicity. This suggests that enantiomer-specific interactions with biological membranes and carriers may influence bioavailability and tissue distribution [23].

### 3.4. Propranolol’s Solubility

Propranolol’s solubility can vary depending on the medium (Table 2). It is classified as a highly soluble drug under the Biopharmaceutics Drug Disposition Classification System (BDDCS Class 1) [24].

Water, with a moderate solubility of approximately 29 mg/mL, is suitable for oral and intravenous formulations of propranolol. This is particularly relevant given propranolol’s classification as a BDDCS Class 1 drug, indicating high solubility and permeability. DMSO and DMF offer very high solubility, making them useful for in vitro studies, although their toxicity limits clinical application. Moreover, high solubility in DMSO allows for the preparation of concentrated stock solutions for cell culture experiments, where propranolol is often used as a β-AR modulator. Micellar and Emulsion-Based Carriers such as Tween 20, Tween 80, and Kolliphor EL are surfactants commonly used in micellar and emulsion-based drug delivery systems. Despite their relatively low solubility values, they can enhance the bioavailability of propranolol in biological environments by improving dispersion and absorption. Organic solvents such as chloroform, ether, benzene, and ethyl acetate exhibit very poor solubility for propranolol, which restricts their use in pharmaceutical formulations.

### 3.5. Pharmacodynamics and Metabolism of Propranolol

Propranolol exerts its therapeutic effects through competitive antagonism of β_1_- and β_2_-ARs [8]. By blocking β_1_-AR in the heart, propranolol reduces heart rate, myocardial contractility, and overall oxygen demand, which is critical in managing ischemic heart conditions such as angina. β_2_ receptor antagonism, located in bronchial and vascular smooth muscle, can cause bronchoconstriction, making propranolol less suitable for asthmatic patients. Additionally, propranolol inhibits renin release from the kidneys, contributing to its antihypertensive action.

Beyond cardiovascular effects, propranolol’s ability to cross the blood–brain barrier [20] allows it to influence central adrenergic pathways, which is beneficial for treating anxiety and migraine. Its central effects are thought to result from dampening the sympathetic nervous system’s response to stress.

Nevertheless, its significant first-pass metabolism in the liver, mediated largely by the cytochrome P450 enzymes (CYP2D6 and CYP1A2) [30], limits its bioavailability to about 25–35% following oral administration [19]. This necessitates the use of higher doses or the development of sustained-release formulations to maintain therapeutic plasma concentrations.

Additionally, propranolol is subject to several metabolic transformations, including aromatic hydroxylation, N-dealkylation, and glucuronidation [31]. These modifications not only reduce the parent drug’s pharmacological activity but also influence its elimination. Despite its relatively low oral bioavailability, propranolol’s metabolism yields both active and inactive metabolites, such as 4-hydroxypropranolol, which possesses significant beta-blocking activity and contributes to the overall therapeutic effects of the drug [19]. Propranolol undergoes extensive hepatic metabolism, primarily through cytochrome P450 enzymes, particularly CYP2D6 [12,32]. Due to significant first-pass metabolism, only a fraction of the orally administered dose reaches systemic circulation, resulting in bioavailability as low as 25% [33]. Propranolol has a plasma half-life of approximately 3–6 h, necessitating multiple daily doses in chronic therapy [34]. However, extended-release formulations have been developed to improve patient compliance [19]. Genetic polymorphisms in CYP2D6 can result in variable metabolism rates, influencing propranolol’s therapeutic efficacy and the risk of adverse effects [35]. Renal excretion is the primary route for eliminating the drug’s metabolites, with less than 1% excreted unchanged [36].

## 4. Crystallographic Aspect of Propranolol

Crystallography, the scientific discipline dedicated to elucidating the atomic arrangement of crystalline materials, plays a pivotal role in understanding the physicochemical properties of substances [37]. Since 2020, this field has experienced substantial advancements in both methodology and practical applications [2]. One notable development is NMR crystallography [18,38], which integrates nuclear magnetic resonance spectroscopy with theoretical calculations to precisely determine crystal structures. This technique is particularly valuable for investigating pharmaceutical crystal forms at the atomic level.

In pharmaceutical research, crystallography provides detailed insights into the molecular structures of chemical compounds and biomolecules, which is essential for drug discovery and design [2,8,39,40]. X-ray crystallography enables the determination of three-dimensional protein structures and their complexes with ligands, deepening our understanding of enzyme and receptor functions—knowledge that is crucial for structure-based drug design [39,41].

X-ray powder diffraction (XRPD) is extensively employed to identify and characterize different crystalline forms of active pharmaceutical ingredients (APIs). Because polymorphism can profoundly affect a drug’s solubility, bioavailability, and stability, controlling these solid-state forms is crucial in pharmaceutical development and manufacturing. XRPD also allows monitoring of structural changes during storage and formulation, ensuring the safety and efficacy of pharmaceutical products [42,43].

It is essential to distinguish between canonical and non-canonical drug targets. Canonical targets refer to primary, well-characterized biological entities—such as receptors, enzymes, or transporters—that a drug is specifically designed to modulate in order to elicit its therapeutic effect. These targets undergo extensive clinical validation and regulatory scrutiny. In the case of propranolol, canonical targets include the β_1_- and β_2_-adrenergic receptors (β_1_- and β_2_-ARs), which mediate its cardiovascular effects.

In contrast, non-canonical targets are secondary or unexpected biological structures that a drug may bind to but are not part of its intended mechanism of action. Such interactions can result in off-target effects, side effects, or even reveal new therapeutic opportunities. Propranolol has been shown to interact with proteins like Cel7A, a cellulase enzyme, and lactoferrin, indicating potential roles beyond cardiovascular therapy. Understanding both canonical and non-canonical targets is essential for optimizing drug efficacy, minimizing adverse effects, and exploring novel applications.

### 4.1. Reported Crystallographic Structures of Propranolol

Table 3 summarizes selected high-resolution crystal structures of proteins obtained from the Protein Data Bank (PDB). These proteins represent diverse biological functions and taxonomic origins, including enzymes involved in polysaccharide degradation, membrane receptors essential for signal transduction, and iron-binding proteins with immunological significance.

Two entries, 1DY4 and 6GRN, correspond to cellobiohydrolase Cel7A from *Trichoderma reesei*, a filamentous fungus widely studied for its cellulolytic activity. These enzymes are key in hydrolyzing cellulose into cellobiose units, and their structures—resolved at 1.8 Å and 1.7 Å, respectively—offer detailed insights into substrate binding and catalytic mechanisms.

The structure 1H46 represents the catalytic module of Cel7D from *Phanerochaete chrysosporium*, another fungus specialized in lignocellulose degradation. Its high resolution of 1.6 Å contributes to understanding the diversity and specialization among fungal cellulases.

Structures 2RH1 and 6PS5 depict the human β_2_-adrenergic receptor (hβ2-AR), a G protein-coupled receptor (GPCR) regulating cardiovascular and pulmonary functions. Resolved at 2.4 Å and 2.8 Å, these structures are crucial for drug design targeting adrenergic signaling pathways, especially relevant for β-blocker pharmacology.

Finally, 3MJN corresponds to the C-lobe of lactoferrin from *Bos taurus*, an iron-binding glycoprotein with antimicrobial and immunomodulatory properties. The 2.38 Å resolution structure reveals detailed iron coordination and potential receptor interactions.

Collectively, these structures span a resolution range of 1.6 Å to 2.8 Å, providing atomic-level insights into protein function, ligand interactions, and structural dynamics. Their inclusion enables comparative analyses across enzymatic, receptor, and transport protein classes and lays a foundation for structure-based drug design and protein engineering.

#### 4.1.1. β-Adrenergic Receptors (β-ARs)

Chromaffin cells in the adrenal medulla release epinephrine (E) and norepinephrine (NE) directly into the bloodstream upon stimulation by sympathetic nerve fibers. Catecholamines like E and NE exert their effects through α- and β-adrenergic receptors (α-, β-ARs) [44], regulating key physiological functions such as blood pressure, heart rate, myocardial contractility, metabolism, and CNS activity [45].

Activation of β-ARs in immune cells initiates signaling cascades, including the β_2_ → cAMP → PKA → Ca^2+^ pathway, as well as non-canonical mechanisms. These pathways often result in immunosuppressive effects, such as inhibition of interleukin-2 (IL-2) secretion and reduced macrophage phagocytic activity [46].

Pharmacological blockade of β-ARs leads to vasoconstriction, downregulation of angiogenic factors like VEGF and bFGF, induction of endothelial cell apoptosis, and suppression of the renin–angiotensin–aldosterone (RAA) system [37]. In the CNS, regions such as the amygdala release epinephrine as a neurotransmitter, forming the locus coeruleus–noradrenergic (LC-NA) system, which regulates arousal, attention, and stress responses. The hypothalamic–pituitary–adrenal (HPA) axis and sympathoadrenergic fibers serve as the main neuroimmune communication pathways [46].

Pharmacological blockade of β-ARs inhibits receptor activation, thereby attenuating the effects of epinephrine and norepinephrine on tissues expressing β-AR subtypes: β_1_-AR: brain, kidneys, lungs, spleen, liver, muscles, salivary glands [47]; β_2_-AR: brain, lungs (airway smooth muscle, epithelial cells, submucosal glands), vascular endothelium, vascular smooth muscle, immune cells (mast cells, macrophages, eosinophils) [48], lymphocytes, skin, liver, heart [49]; β_3_-AR: adipose tissue, stomach, gallbladder, blood vessels in cancer and immune cells; β_4_-AR and β_1_-AR isoforms: adipose tissue, heart. β_1_-AR activation reduces COX2 levels and promotes its degradation [50].

Emerging evidence suggests that β-ARs are densely distributed in the nervous system, including peripheral nociceptors and spinal cord regions, and may influence pain perception [51]. Although the exact mechanisms by which β-blockers alleviate pain remain unclear, β_2_-ARs are known to be expressed in nociceptive pathways of both the central and peripheral nervous systems [52]. β-ARs belong to the G protein-coupled receptor (GPCR) family and are essential components of the sympathetic nervous system, mediating responses to catecholamines and regulating circulatory, respiratory, and metabolic functions. Both β_1_- and β_2_-ARs are present in the heart, but they perform distinct roles in cardiac regulation.

**Figure 2:**
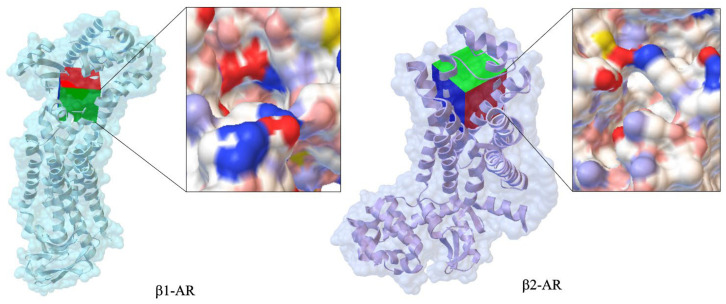
Comparison of crystallographic structures of canonical propranolol’s targets such as β_1_-AR (PDB ID: 7BU6, 2.80 Å) and β_2_-AR (PDB ID: 1RH1, 2.40 Å).

Figure 2 compares the crystallographic structures of β_1_-AR (PDB ID: 7BU6, 2.80 Å) and β_2_-AR (PDB ID: 1RH1, 2.40 Å). These structures help explain drug selectivity and binding mechanisms. Differences in ligand affinity between β_1_ and β_2_ receptors are largely due to variations in their extracellular vestibules, which are critical for ligand entry and receptor specificity and association kinetics. The electrostatic surface map of β_1_-AR reveals a continuous negatively charged tunnel, while β_2_-AR features two negatively charged zones separated by a neutral gap, potentially influencing ligand orientation and stabilization.

Despite similar catecholamine-binding pockets, the distinct vestibule shapes and electrostatic properties influence how norepinephrine and β-blockers reach the orthosteric site, affecting association rates, binding affinities, and receptor activation thresholds.

Propranolol binds to β_1_-AR and β_2_-AR in a similar manner, but differences in the extracellular domain and transmembrane helices contribute to its selectivity. In β_1_-AR, propranolol forms hydrogen bonds with residues such as Asp121 and hydrophobic contacts with Val117, which help stabilize the receptor’s inactive conformation [8].

Recent mutagenesis and molecular modeling studies have shown that transmembrane helices III, V, and VI—particularly residues like Asn310 in β_1_-AR and Tyr308 in β_2_-AR—play a pivotal role in subtype-specific ligand recognition. These non-conserved residues modulate hydrogen bonding and π–π stacking interactions with propranolol’s aromatic ring system, contributing to its differential affinity.

**Figure 3:**
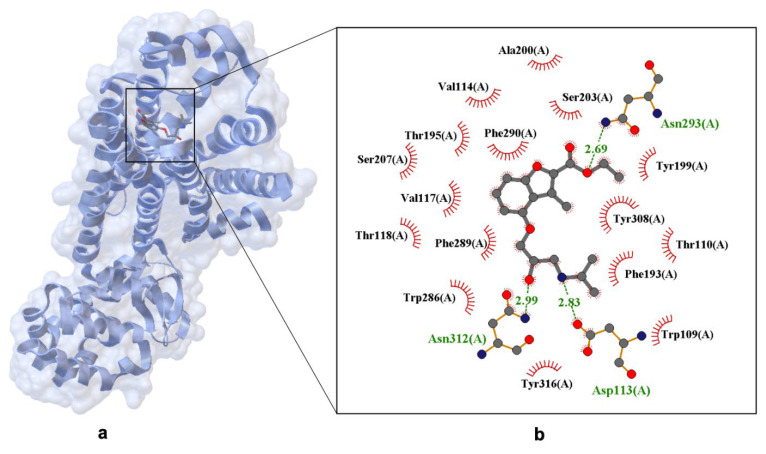
X-ray structure of propranolol bind in the binding site of the human β_2_-adrenergic Receptor (hβ_2_-AR) crystal. Panel (**a**) shows the overall receptor-ligand complex. (PDB ID: 3NY9, 2.70 Å [54]). Panel (**b**) presents an enlarged view of the ligand-binding site. Hydrogen bonds are depicted as dashed lines with interatomic distances labeled in Å, and Van der Waals interactions are indicated by labeled arcs with radial spokes pointing toward the ligand atoms.

A key structure illustrating propranolol’s binding is human β_2_-AR bound to carazolol (PDB ID: 5X7D) [53], which serves as a close analog for propranolol interaction. Another important structure is β_2_-AR bound to propranolol (PDB ID: 3NY9), showing how the drug forms hydrogen bonds and stabilizes aromatic moieties, reinforcing its inverse agonist activity (Figure 3) [54].

Figure 3 highlights key amino acid residues involved in ligand recognition and stabilization, including hydrogen bonds: Ser207(A), Tyr199(A), Asn312(A) (distances: 2.69 Å, 2.83 Å, 2.99 Å); aromatic interactions: Trp286(A), Phe289(A), Tyr308(A), Tyr316(A); polar contacts: Thr110(A), Thr118(A), Asp113(A); hydrophobic contacts: Val117(A), Trp109(A). This complex network of interactions explains propranolol’s high affinity for both β_1_ and β_2_ receptors, while subtle structural differences account for variations in binding specificity.

Electrostatic mapping and crystallographic overlays further confirm that propranolol’s amphiphilic nature—combining a hydrophobic naphthalene ring with polar functional groups—enables it to engage both hydrophobic pockets and polar residues, enhancing its binding versatility across receptor subtypes.

#### 4.1.2. The Interaction Between Propranolol and the Cel7A Enzyme

The interaction between propranolol and the enzyme Cellobiohydrolase Cel7A represents a case of non-canonical drug targeting, illustrating its unexpected enzymatic binding behavior. Cel7A plays a crucial role in cellulose degradation, a key step in biofuel production from plant biomass [55].

The X-ray crystal structure of propranolol bound to Cel7A (PDB ID: 1DY4) offers valuable insights into this interaction, particularly the enantioselective binding of the (S)-propranolol enantiomer. The structure was resolved at 1.8 Å, allowing for atomic-level visualization of molecular interactions within the enzyme’s active site.

**Figure 4:**
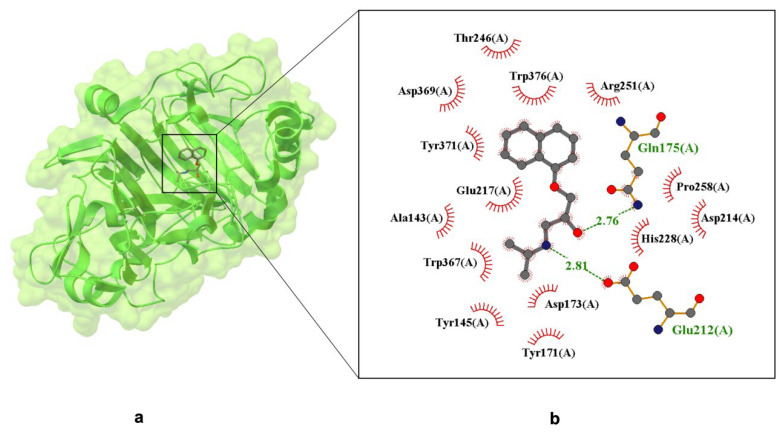
X-ray crystal structure of (S)-propranolol within the active site of Cellobiohydrolase Cel7A (formerly CBH I), resolved at 1.80 Å (PDB ID: 1DY4) [51]. Panel (**a**) displays the overall protein–ligand complex and a magnified view of the binding pocket. Panel (**b**) highlights Van der Waals interactions, depicted by labeled arcs with radial spokes pointing toward the ligand atoms. Hydrogen bond-forming residues are shown in ball-and-stick format, with dashed lines indicating interactions and interatomic distances labeled in Å.

This structure clearly differentiates between the two propranolol enantiomers, with the (S)-enantiomer specifically occupying the binding pocket. A key finding is the preferential binding of (S)-propranolol, which fits into the active site with a defined orientation. The interaction is primarily stabilized by hydrogen bonds, hydrophobic contacts, and π–π stacking interactions (see Figure 4) [56].

Figure 4 illustrates hydrogen bonds between propranolol and the following residues: Gln175(A)—2.81 Å; Glu212(A)—2.76 Å (these are marked in green). Red semicircles around the ligand indicate residues involved in hydrophobic interactions, including Trp367(A), Trp376(A), Tyr145(A), Tyr171(A), Tyr371(A)—aromatic residues capable of π–π stacking; Ala143(A), Pro258(A)—hydrophobic residues contributing to ligand stabilization. Additionally, Glu212 and Glu217 form a bidentate salt bridge with the protonated secondary amine of propranolol, a critical interaction for binding specificity and enantioselectivity. Asp214, located within 3.7 Å, may further stabilize the ligand via electrostatic attraction. These findings confirm that propranolol is anchored in the binding pocket through a combination of hydrogen bonding, π-stacking, and electrostatic forces. At pH 7.0, Asp214 and His228 modulate the local charge environment, enhancing the drug-protein interaction [57]. This evidence supports the hypothesis that propranolol can bind stably to non-canonical targets, potentially affecting its pharmacokinetics and off-target activity.

**Figure 5:**
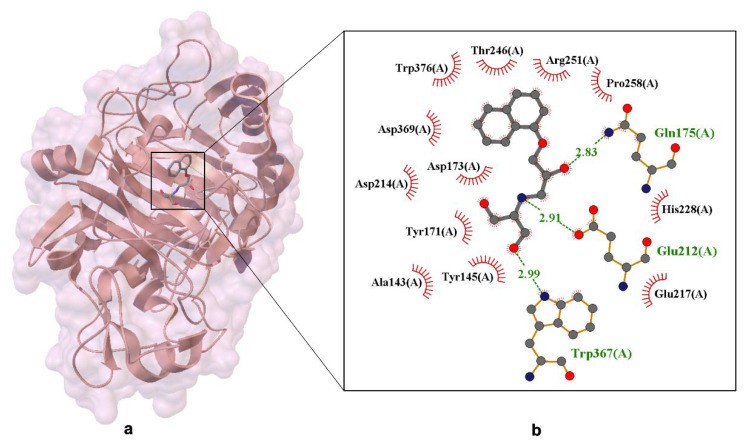
X-ray crystal structure of dihydroxy-propranolol ((R/S)-DHP R2/S2) bound within the active site of Cellobiohydrolase Cel7A. Panel (**a**) shows the overall protein–ligand complex, while panel (**b**) presents an enlarged view of the ligand-binding pocket. Residues forming hydrogen bonds with the ligand are depicted in ball-and-stick representation, with dashed lines indicating interactions and interatomic distances labeled in angstroms (Å). Van der Waals interactions are illustrated by labeled arcs with radial spokes pointing toward the ligand atoms. The structural data provide insight into the binding mode of propranolol metabolites within a non-canonical enzymatic target, suggesting potential off-target interactions and contributing to the understanding of ligand–enzyme recognition mechanisms [18].

In contrast, the (R)-propranolol enantiomer (PDB ID: 6GRN, resolved at 1.79 Å) exhibits weaker binding to Cel7A due to steric hindrance and lower affinity for the active site [58]. The ligand’s binding primarily involves interactions with the enzyme’s catalytic residues, including Glu212, Glu217, and Asp214, which are essential for propranolol binding (see Figure 5).

The distances shown in the figure (approximately 2.83 Å and 2.91 Å) emphasize the precise geometry of these interactions, which are essential for binding specificity and overall ligand stability. Additionally, side-chain interactions occur between the ligand and residues such as Trp376, Trp367, and Tyr171, forming a stabilizing network. These residues belong to the glucosyl-binding platform, which is vital for maintaining the structural integrity and catalytic efficiency of the enzyme.

The combination of favorable hydrogen bonds and Van der Waals interactions highlights the entropy-driven nature of propranolol’s binding to Cel7A. This thermodynamic profile suggests that ligand binding is governed not only by enthalpic contributions but also by conformational flexibility and solvent displacement.

The X-ray crystal structure of the Cel7A–propranolol complex provides crucial atomic-level insights into the binding mechanism. Crystallographic analysis revealed that (S)-dihydroxypropranolol (S2) binds in a similar position to (S)-propranolol, with the naphthyl group stacking against Trp376, and the secondary amine interacting with Glu212 and Glu217. Moreover, the hydroxymethyl groups of S2 form specific hydrogen bonds with Trp367 and Glu217, further reinforcing the ligand-protein interaction.

The high-resolution data at 1.79 Å enables precise determination of these interactions and the overall geometry of the binding site. It reveals that the dihydroxy analogue binds more tightly than propranolol, owing to these additional stabilizing interactions.

The hydrogen bonds observed in the S2 complex provide a structural basis for the increased affinity of the dihydroxy derivative, supporting the idea that ligand modifications can substantially influence both affinity and selectivity in enzyme-ligand interactions.

The (S)-enantiomer of propranolol is preorganized for binding, resulting in stronger interactions and a more stable complex compared to the (R)-enantiomer. Furthermore, structural modifications, such as the addition of hydroxymethyl groups, enhance both binding affinity and selectivity. Among these, the dihydroxy derivative (S2) exhibits the strongest binding, due to its additional hydrogen bonding and improved complementarity. These findings offer valuable insights into the molecular basis of enantioselectivity and support the rational design of new ligands with enhanced affinity and specificity for Cel7A [18].

**Figure 6:**
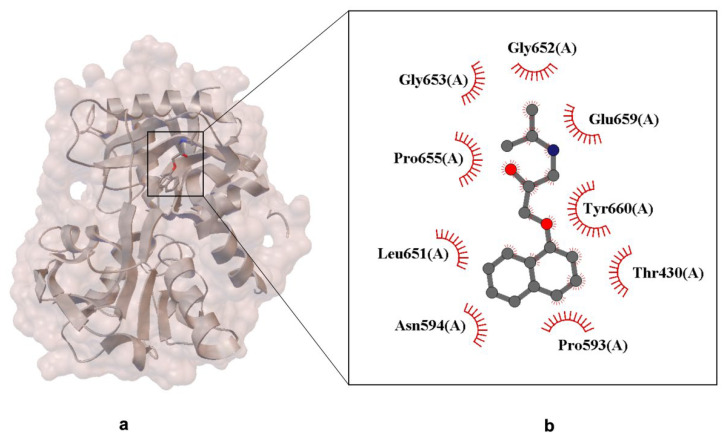
X-ray crystal structure of (R)-propranolol bound to the C-lobe of lactoferrin (PDB ID: 3MJN, resolution 2.38 Å). Panel (**a**) shows the overall protein–ligand complex. Panel (**b**) presents a close-up of the ligand-binding site. The propranolol molecule is represented in ball-and-stick format. Residues involved in Van der Waals interactions with the ligand are indicated by labeled arcs with radial spokes pointing towards the ligand atoms [39].

The structural data presented in Figure 6 reveal the binding of (R)-propranolol within the C-lobe of lactoferrin, an iron-binding glycoprotein known for its antimicrobial and immunomodulatory functions.

The crystallographic image highlights the molecular interactions between propranolol and neighboring protein residues (Figure 6). The non-canonical binding site is located in a pocket formed by the protein’s β-sheet and α-helix secondary structures, which accommodate the ligand through a combination of spatial complementarity and chemical compatibility. Specific residues interacting with propranolol include Gly652, Glu659, Pro655, and Tyr660, with interactions represented by red curly arrows. These likely involve hydrogen bonds, Van der Waals forces, and hydrophobic contacts, typical of small molecule–protein binding. The stabilization of the ligand appears to be driven primarily by dispersion forces and steric complementarity, rather than strong electrostatic interactions. This is consistent with propranolol’s amphiphilic nature, which allows it to interact with both polar and nonpolar residues within the binding pocket.

The presence of a mixed polarity environment creates a versatile interaction platform, enabling propranolol to engage with non-canonical protein targets such as lactoferrin. This may contribute to its pleiotropic effects and off-target pharmacology, including immunomodulatory and neuroprotective roles reported in recent studies. From a drug design perspective, these findings emphasize the importance of evaluating ligand promiscuity and considering protein–ligand interactions beyond primary therapeutic targets, especially when assessing metabolite behavior, tissue distribution, and unintended binding events.

**Figure 7:**
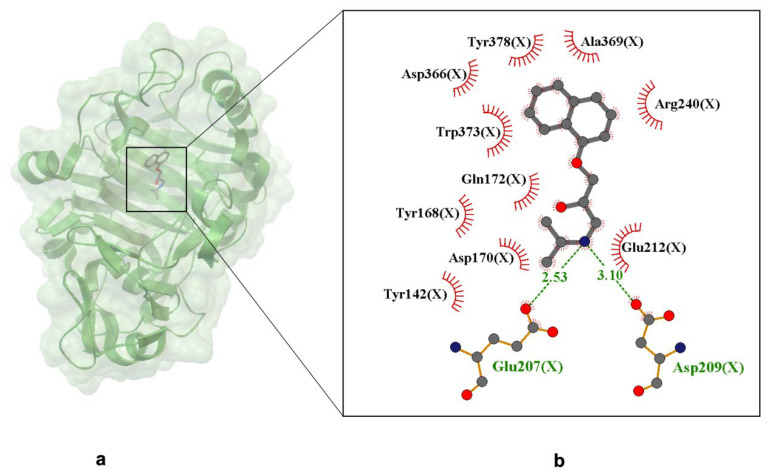
X-ray crystal structure of (S)-propranolol bound to the C-lobe of lactoferrin (PDB ID: 1H46, resolution 1.06 Å). Panel (**a**) shows the overall protein–ligand complex. Panel (**b**) presents an enlarged view of the ligand-binding site. Hydrogen bonds are depicted as dashed lines with interatomic distances labeled in Å, and Van der Waals interactions are indicated by labeled arcs with radial spokes [52].

The crystallographic analysis of the catalytic module of Cel7D from Phanerochaete chrysosporium complexed with (R)-propranolol reveals detailed interactions governing binding and enantioselectivity. The (R)-propranolol molecule occupies the enzyme’s active site, specifically the −1/+1 glucosyl-binding subsites, interacting with key cata-lytic and substrate-binding residues (Figure 7). The ligand’s secondary amine group forms a strong charge–charge interaction with Glu207(X), the catalytic nucleophile, at a distance of 2.53 Å. Additionally, Asp209(X) stabilizes the ligand by interacting with the amine group. These electrostatic interactions are essential for ligand affinity and contribute to enantioselective recognition. The alcohol group of propranolol forms a hydrogen bond with a water molecule, while the ether oxygen does not engage in direct hydrogen bonding with the enzyme. Hydrophobic interactions occur between the ligand’s aromatic naphthyl group and the indole ring of Trp373(X), as well as side chains of Tyr142(X), Tyr168(X), and Ala369(X), stabilizing the ligand within the bind-ing cleft Notably, the naphthyl group stacks onto Trp373(X), establishing π–π interactions that further reinforce binding. The structure shows that (R)-propranolol binds similarly to other ligands in the substrate-binding cleft, competing with inhibitors like cellobiose and lactose, which bind in the +1 and +2 subsites, thereby interfering with propranolol binding. These interactions provide a structural basis for the observed inhibitory effect of (R)-propranolol on Cel7D. Further stabilization occurs through Van der Waals contacts with residues such as Tyr378(X), Asp366(X), Arg240(X), and Gln172(X), contributing to shape complementarity and enhanced binding affinity.

A comparison of binding modes between (R)-propranolol in Cel7D and (S)-propranolol in Cel7A from *Trichoderma reesei* reveals distinct enantioselectivity differences. While both enzymes bind their respective enantiomers at analogous active site positions, structural variations in the loop regions surrounding the binding site explain the different binding preferences. In Cel7D, the less enclosed active site structure allows (R)-propranolol to bind more tightly than (S)-propranolol, supported by interactions involving the naphthyl group and the spatial arrangement of neighboring residues.

These findings underscore the significance of structural variations in the enzyme’s substrate-binding cleft for the enantioselective recognition of propranolol and related chiral compounds. Propranolol’s ability to interact with non-canonical enzymatic targets like Cel7D broadens its interaction landscape, potentially impacting its pharmacological profile and therapeutic versatility. Understanding these off-target interactions is critical to optimizing drug selectivity, minimizing adverse effects, and guiding the rational design of next-generation β-blockers or propranolol analogs with tailored binding properties.

Beyond its well-established interactions with β_1_- and β_2_-ARs, propranolol demonstrates binding affinity to non-canonical proteins such as the cellulase enzyme Cel7A and the iron-binding glycoprotein lactoferrin. These interactions, while not part of propranolol’s primary therapeutic mechanism, may have significant implications for its broader pharmacological profile.

The binding of propranolol to Cel7A, a fungal cellulase involved in polysaccharide degradation, suggests potential off-target effects in microbial or enzymatic environments. Although the physiological relevance of this interaction remains speculative, it raises intriguing possibilities regarding propranolol’s influence on microbiota or its behavior in biotransformation pathways. Similarly, the interaction with lactoferrin—a protein known for its immunomodulatory and antimicrobial properties—may contribute to propranolol’s observed effects in inflammatory or immune-related conditions.

These non-canonical interactions support the hypothesis that propranolol’s amphiphilic character and electrostatic surface distribution enable it to engage with a diverse array of biological targets. This molecular promiscuity could underlie some of its off-label applications, such as in the treatment of infantile hemangiomas, post-traumatic stress disorder (PTSD), and certain cancers, where immunological and angiogenic pathways are involved.

Future studies should explore whether these off-target bindings are pharmacologically active or merely incidental. Structural modeling and binding affinity assays could help determine whether such interactions modulate protein function or contribute to therapeutic or adverse effects. Understanding these mechanisms may open new avenues for drug repurposing and inform the design of β-blockers with tailored target profiles.

#### 4.1.3. Interaction Between Propranolol and the Human β_2_-Adrenergic Receptor

**Figure 8:**
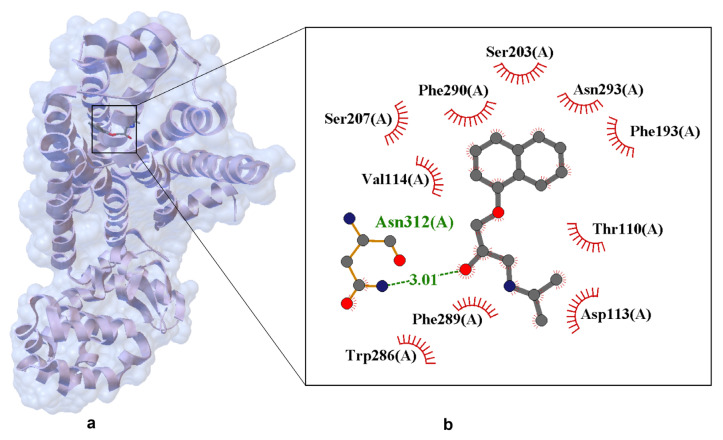
Experimentally resolved X-ray crystal structure of propranolol bound to the human β_2_-adrenergic receptor (PDB ID: 6PS5, resolution 2.80 Å) [59]. Structure obtained using serial femtosecond crystallography (SFX) at an XFEL source, enabling high-resolution data collection from microcrystals under ambient conditions. Panel (**a**) shows the overall protein–ligand complex. Panel (**b**) presents a close-up of the ligand-binding site. Hydrogen bonds are shown as dashed lines with interatomic distances labeled in angstroms (Å). Van der Waals interactions are indicated by labeled arcs with radial spokes pointing toward the ligand atoms. Key interacting residues include Hydrogen bonding: Ser203, Ser207, Asn293, Asp113; Van der Waals contacts: Phe193, Val114, Thr110, Phe289, Phe290, Trp286, Asn312.

Propranolol binds to the hβ_2_-AR in a canonical antagonist-binding pose, as revealed by the experimentally determined X-ray crystal structure (PDB ID: 6PS5). In this structure, the ethanolamine moiety of propranolol forms hydrogen bonds with Asp113(A) and Asn312(A), key residues within the orthosteric binding pocket (Figure 8). The 6PS5 is the only available crystal structure of propranolol bound to its canonical target (β_2_-AR). The structure was obtained using serial femtosecond crystallography (SFX) at an X-ray Free Electron Laser (XFEL) facility, as described by Ishchenko et al. [59], and therefore qualifies as a crystal structure rather than a computational model.

The crystallographic structure of hβ_2_-AR in complex with propranolol provides a high-resolution view of the molecular determinants underlying β-blocker binding and antagonism. Propranolol is positioned within the orthosteric site, engaging in a network of stabilizing interactions that collectively inhibit receptor activation. A key hydrogen bond is formed between the ligand’s secondary amine and Asp113(A), with a bond length of 3.01 Å, anchoring propranolol in a conformation that sterically and electrostatically blocks agonist access. Additional polar contacts with Ser203(A), Ser207(A), Asn293(A), and Asn312(A) reinforce ligand stabilization, while hydrophobic residues such as Phe290(A), Phe289(A), Phe193(A), Trp286(A), Val114(A), and Thr110(A) contribute to shape complementarity and dispersion forces. Notably, the naphthalene ring of propranolol engages in π–π stacking interactions with aromatic residues, enhancing the ligand’s residence time and binding affinity. These interactions reflect a conserved binding motif among β_2_-AR antagonists, consistent with structurally related compounds such as alprenolol and timolol.

Importantly, the structure was obtained using serial femtosecond crystallography (SFX) at an XFEL facility, a technique that enables the collection of diffraction data from microcrystals using ultrashort X-ray pulses on the femtosecond scale. This approach allows for high-resolution structural determination before radiation damage occurs. In the case of 6PS5, propranolol was introduced into preformed crystals via ligand exchange, replacing timolol used during initial crystallization. The resulting structure captures propranolol in its native binding conformation under near-physiological conditions, without the need for cryogenic cooling.

Minimal conformational changes observed upon ligand binding are characteristic of competitive antagonists, which occupy the orthosteric site without triggering the conformational rearrangements required for G protein activation. Electron density maps from the 6PS5 structure confirm the successful exchange of transient crystallization ligands with propranolol, validating the structural integrity of the complex and supporting its relevance as a model for structure-based drug design.

#### 4.1.4. Comparative Structural Analysis of Selected β-Blockers

**Figure 9:**
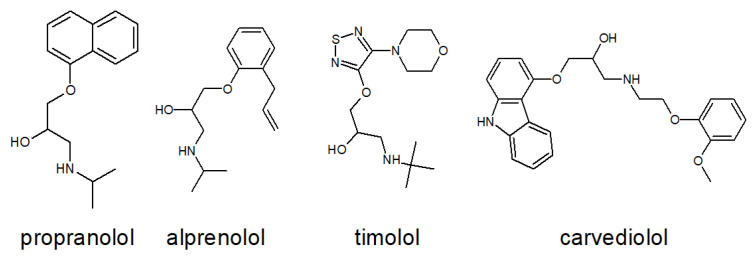
Structural comparison of four β-blockers: propranolol, alprenolol, timolol, and carvedilol. The figure illustrates similarities and differences in their chemical frameworks, particularly in functional groups that may influence receptor binding and pharmacological activity.

Figure 9 presents a comparative structural analysis of four clinically relevant β-blockers: propranolol, alprenolol, timolol, and carvedilol. The figure highlights both conserved and divergent features in their chemical frameworks, with particular emphasis on functional groups that influence receptor binding, pharmacological activity, and therapeutic application.

All four compounds share a conserved propanolamine side chain (–CH(OH)–CH_2_–NH–), which is critical for anchoring the ligand within the orthosteric site of β-ARs. This moiety facilitates hydrogen bonding and electrostatic interactions with key residues such as Asp113 and Asn312, as demonstrated in the crystal structure of propranolol bound to hβ_2_-AR (Figure 8). Despite this shared pharmacophore, each β-blocker exhibits distinct structural features that shape its pharmacodynamic and pharmacokinetic profile: Propranolol contains a rigid naphthalene ring, which increases lipophilicity and enhances CNS penetration, making it suitable for systemic and neuropsychiatric indications. Alprenolol features a methyl-substituted phenyl ring, resulting in slightly modified receptor affinity and altered metabolic stability. Timolol incorporates a thiadiazole ring and a morpholine-like ether bridge, increasing hydrophilicity and favoring topical ophthalmic use, particularly in the treatment of glaucoma. Carvedilol is distinguished by its carbazole moiety and phenolic ring, which confer dual activity as a β-blocker and α_1_-adrenergic antagonist. Additionally, carvedilol exhibits β-arrestin-biased signaling, contributing to its unique cardioprotective effects beyond classical receptor blockade.

To further quantify the structural similarity between propranolol and the other β-blockers, a superimposition analysis was performed. The resulting Root Mean Square Deviation (RMSD) values provide insight into the degree of spatial overlap between molecular frameworks: Propranolol–alprenolol: RMSD = 0.032 Å, indicating very high structural similarity, particularly in the orientation of the propanolamine chain and aromatic ring system. This close resemblance supports their comparable receptor binding profiles and overlapping pharmacological effects. Propranolol–carvedilol: RMSD = 0.078 Å, suggesting moderate similarity, with conserved backbone geometry but notable differences in the extended aromatic system and additional functional groups responsible for α_1_-blocking and biased signaling. Propranolol–timolol: RMSD = 1.078 Å, reflecting significant structural divergence, primarily due to the presence of the thiadiazole ring and ether bridge in timolol, which alter its three-dimensional conformation and physicochemical properties. These RMSD values reinforce the qualitative observations from Figure 9 and highlight how even subtle chemical modifications can lead to pronounced differences in molecular geometry, receptor interaction potential, and clinical application. The close alignment between propranolol and alprenolol may explain their similar β_1_/β_2_ selectivity and systemic use, while the distinct geometry of timolol correlates with its hydrophilic profile and suitability for ocular delivery.

Taken together, the insights from Figure 8 and Figure 9—supported by superimposition analysis—provide a robust foundation for the rational design of next-generation β-adrenergic ligands. Such compounds may offer enhanced receptor selectivity, optimized pharmacokinetics, and reduced off-target effects, ultimately improving clinical outcomes in cardiovascular, ophthalmic, and CNS-related disorders [59].

## 5. Conclusions

This study offers a multidimensional perspective on propranolol by integrating pharmacological profiling with crystallographic analysis, revealing how molecular structure informs therapeutic behavior. The inclusion of the high-resolution structure PDB ID: 6PS5—currently the only experimentally resolved crystal of the hβ_2_-AR bound to propranolol—provides a physiologically relevant snapshot of ligand–receptor interactions. This structural insight enables precise identification of binding determinants responsible for propranolol’s antagonistic activity.

The comparative superimposition of propranolol with other β-blockers (alprenolol, timolol, carvedilol) demonstrates measurable differences in molecular geometry that correlate with receptor selectivity and pharmacokinetic profiles. These findings underscore the pharmacological relevance of propranolol’s amphiphilic character, stereochemistry, and electrostatic properties, as revealed through crystallographic and comparative analyses.

A key focus of this review is the enantioselective behavior of propranolol. The S-enantiomer exhibits significantly higher receptor affinity and pharmacological potency than the R-form, reinforcing the rationale for enantiomer-specific formulations and prompting reconsideration of racemic dosing strategies in clinical practice.

Importantly, propranolol’s ability to interact with non-canonical targets such as Cel7A and lactoferrin expands its pharmacological landscape beyond cardiovascular applications. These off-target interactions suggest potential roles in immunomodulation, oncology, and neuroprotection, opening avenues for drug repurposing.

Overall, this work highlights the value of an interdisciplinary approach in drug research. By combining structural biology, pharmacodynamics, and ligand modeling, we gain a deeper understanding of propranolol’s molecular behavior and therapeutic versatility. These insights support the rational design of next-generation β-adrenergic ligands with improved selectivity, reduced side effects, and broader clinical utility—contributing to the evolving paradigm of precision medicine. Future studies should explore enantiomer-specific formulations and investigate propranolol’s off-target interactions for potential therapeutic repurposing.

## Figures and Tables

**Table 1:** Pharmacokinetic profile of propranolol [19].

Parameter	Description
Absorption	Rapid and complete absorption, peak plasma concentrations in 1–3 h.
Bioavailability	~26%, due to significant first-pass hepatic metabolism
Protein Binding	>90%, primarily bound to albumin
Half-Life	3–6 h, requiring multiple daily doses
Metabolism	Extensive hepatic metabolism via cytochrome P450 enzymes, forming active metabolite 4-hydroxypropranolol
Excretion	<1% excreted unchanged in urine, indicating extensive biotransformation
Volume of distribution (Vd)	large Vd (~3–5 L/kg), reflecting its extensive tissue binding, especially in lipid-rich organs like the brain and liver.

**Table 2:** Solubility of propranolol in various solvents.

Solvent	Solubility (mg/mL)	Temperature	Reference
Water	~29	37 °C	[25,26]
Ethanol	10	Not specified	[25,26]
DMSO	59	25 °C	[26,27]
DMF	50	Not specified	[26,28]
Tween 80	1.89	37 °C	[29]
Tween 20	1.86	37 °C	[29]
Kolliphor EL	1.32	37 °C	[29]
Chloroform	Slightly soluble	Not specified	[25,26]
Ether	Practically insoluble	Not specified	[25,26]
Benzene	Practically insoluble	Not specified	[25,26]
Ethyl Acetate	Practically insoluble	Not specified	[25,26]

**Table 3:** Crystal structures of propranolol-bound and related proteins from the Protein Data Bank (PDB), illustrating canonical and non-canonical target interactions.

PDB ID	Target	Resolution	Organism	Reference
1DY4	Cellobiohydrolase Cel7A	1.8 Å	Trichoderma reesei	https://www.rcsb.org/structure/1dy4 (accessed on 10 October 2025)
6GRN	Cellobiohydrolase I (Cel7A)	1.7 Å	Trichoderma reesei	https://www.rcsb.org/structure/6GRN (accessed on 10 October 2025)
1H46	Catalytic Module of Cel7D	1.6 Å	Phanerochaete chrysosporium	https://www.rcsb.org/structure/1H46 (accessed on 10 October 2025)
2RH1	β_2_-adrenergic receptor	2.4 Å	Homo sapiens	https://www.rcsb.org/structure/2RH1 (accessed on 10 October 2025)
6PS5	β_2_-adrenergic receptor	2.8 Å	Homo sapiens	https://www.rcsb.org/structure/6ps5 (accessed on 10 October 2025)
3MJN	C-lobe of lactoferrin	2.38 Å	Bos taurus	https://www.rcsb.org/structure/3MJN (accessed on 10 October 2025)

## Data Availability

The original contributions presented in this study are included in the article. Further inquiries can be directed to the corresponding author(s).
